# Coagulation Abnormalities in Dogs with Parvoviral Enteritis

**DOI:** 10.3390/vetsci10010041

**Published:** 2023-01-05

**Authors:** Francesca Corda, Isabella Ballocco, Andrea Corda, Alessandra Mollica, Anna Cilano, Marta Polinas, Maria Luisa Pinna Parpaglia

**Affiliations:** Department of Veterinary Medicine, Veterinary Teaching Hospital, University of Sassari, 07100 Sassari, Italy

**Keywords:** hemostatic disorder dog, parvoviral enteritis, canine parvovirus, CPV-2, coagulation test, systemic inflammatory response syndrome

## Abstract

**Simple Summary:**

Canine parvoviral enteritis is a common cause of morbidity and mortality in young dogs worldwide. Systemic inflammatory response as well as the loss of endogenous anticoagulant, and the vascular endothelial damage, often predispose dogs to a hypercoagulable state. In the present study, the standard coagulation parameters and the relationship between them and clinical variables were measured in nine dogs affected by parvoviral enteritis. All the dogs included in the study presented alterations of the standard coagulation parameters. Moreover, the study evidenced a linear relationship between the activated partial thromboplastin time and clinical score, underlining the importance of assessing the coagulation parameters in canine parvoviral enteritis.

**Abstract:**

Hemostatic alterations have been documented in dogs with canine parvoviral enteritis. This study’s aims were to measure the standard coagulation parameters, and to assess the relationship between them and the clinical variables in dogs with canine parvoviral enteritis. Nine client-owned dogs with a canine parvoviral infection were included in a prospective, observational clinical study. Clinical score and coagulation status were assessed at admission. All nine dogs showed alterations of three or more standard coagulation variables. A correlation analysis evidenced a significantly high positive correlation between the activated partial thromboplastin time and clinical score. The present study concurs that dogs with canine parvoviral enteritis have coagulation disorders that are detectable by measuring the standard coagulation parameters.

## 1. Introduction

Canine parvovirus enteritis (CPE), caused by the three variants of canine parvovirus type 2 (CPV-2), is one of the most virulent and common enteric diseases in young dogs worldwide [1,2,3]. Canine parvovirus type 2 has a tendency to infect rapidly dividing cells of the gastrointestinal tract, lymphoid tissue, myocardium, and bone marrow, causing hemorrhagic diarrhea, vomiting, severe leukopenia, immunosuppression, and myocarditis [2,4]. Bacterial and endotoxin translocation due to intestinal epithelial damage, and severe immunosuppression may induce the development of systemic inflammatory response syndrome (SIRS), sepsis, and death [2,5]. Systemic inflammation and a reduction in antithrombin III (AT III) activity induce a state of hypercoagulability that, when associated with blood stasis and vascular endothelial damage, may lead to thrombosis and/or disseminated intravascular coagulation (DIC), worsening the prognosis. The AT III activity decrease is secondary to the loss of gastrointestinal wall integrity, severe diarrhea, and endotoxin-mediated activation of coagulation [2,6,7]. Two previous studies have evidenced coagulation disorders in dogs with CPE by using thromboelastography (TEG) and standard coagulation parameters [6,7]. Thromboelastography is a viscoelastic test used to assess global hemostasis by measuring clot formation, clot strength, and fibrinolysis [8,9,10]. Conventional coagulation tests such as prothrombin time (PT), activated partial thromboplastin time (aPTT), fibrinogen concentration, fibrinogen degradation products (FDPs) concentration, D-dimer assays, and AT III activity are routinely used in the clinical evaluation of dogs with systemic disease, all of which have the advantages of being cheaper than TEG and not requiring specialized operators.

The objectives of the present study were to measure the standard coagulation parameters and to assess the relationship between them and the clinical variables in dogs with CPE.

## 2. Materials and Methods

A prospective observational study was carried out from January to March 2019, at the Veterinary Teaching Hospital (VTH) of the University of Sassari, enrolling client-owned puppies with naturally acquired CPE. Dogs with clinical signs consistent with CPE (lethargy, vomiting, and diarrhea or some combination of these) and who tested positive on a fecal PCR analysis (Taq DNA Polymerase kit, Qiagen, Milan, Italy) or by fecal ELISA antigen test (SNAP Parvo Test, IDEXX Laboratories Inc., Westbrook, ME 04092, USA) confirmed by fecal PCR analysis, were included in the study. Dogs were not included in the study if they had comorbidities that could confound outcomes, such as intussusception, concurrent canine coronavirus infection, or parasitosis commonly diagnosed in the Mediterranean region [11,12,13]. Coronavirus infection was excluded by fecal PCR analysis [11], while parasitosis was excluded by copro-microscopic examination, as previously described [11,12,13]. Dogs were also excluded if they had received any medication known to interfere with hemostasis, such as corticosteroids, non-steroidal anti-inflammatory drugs, or anticoagulant dugs, in the last two weeks before the VTH presentation. At admission, baseline data were obtained from each dog including age, sex, breed, clinical signs, medical history, and physical examination findings. Moreover, blood and fecal samples were collected from each dog. Complete blood counts (Mindray BC-2008Vet, Mindary Medical Italy S.R.L., 20090 Trezzano sul Naviglio, Italy), blood gas analysis (Stat Profile Prime CCS Analyzer, Nova Biomedical, Waltham, MA 02454, USA), coagulation panel (STA Compact Max coagulometer, Stago, 92600 Asnières-sur-Seine, France), and blood smear were immediately performed. The coagulation panel consisted of the following parameters: PT, aPTT, fibrinogen concentration, FDPs, D-dimer, and AT III activity. The alteration of the coagulation parameters was arbitrarily defined as mild if the deviation was <25%, moderate if it was ≥25, and <50%, severe if it was ≥50% and <100%, and very severe if it was ≥100%. Disease severity was scored from 0 to 12 according to a clinical score previously described in dogs with CPE [14] (Table 1).

Systemic inflammatory response syndrome was diagnosed if dogs fulfilled at least two of the following four criteria: body temperature <37.8 °C or >39.4 °C, heart rate >140 beats per minute, respiratory rate >30 breaths per minute, or pCO_2_ <32 mmHg (venous or arterial), white blood cells count (WBC) <6000 or >16,000 cells/μL, or >3% band neutrophils [5,15]. All the dogs were hospitalized in the VTH infectious unit and, based on the clinical and analytical results, they underwent therapies aimed at reducing the disorders resulting from the disease such as dehydration, acid-base disorders, electrolytic imbalance, hypoglycemia, and sepsis.

### Statistical Analysis

The statistical analysis was performed using Stata 14 (Stata Corp LLC, 4905 Lakeway Drive College Station, TX, USA). Qualitative variables were described with absolute and relative frequencies, whereas quantitative variables were summarized with means and standard deviations (SD) or medians and ranges for parametric and non-parametric distributions, respectively. The Shapiro–Wilk normality test was used to assess the distribution of the quantitative variables. Spearman’s rank correlation test was used to measure the degree of association between coagulation parameters and clinical score. The correlation coefficient (r) was interpreted as previously described [16]. Statistical significance was considered when *p* < 0.05.

## 3. Results

Nine dogs (six males (67%), and three females (33%)) met the inclusion criteria during the study period. The dog’s signalment and clinical score, as well as the presence or absence of SIRS, are reported in Table 2.

Canine parvovirus infection was diagnosed by fecal PCR analysis in all nine dogs and by a fecal ELISA antigen test plus PCR analysis in six dogs. The clinical score ranged between 4 and 10, and five dogs (56%) met the SIRS criteria. Four of five dogs (80%) with SIRS presented the higher clinical score (between 7 and 10).

Blood gas analysis, obtained at the time of admission, evidenced compensated metabolic acidosis in eight dogs (89%); only dog No. 1 had mixed disorders. Seven dogs (78%) had hyponatremia, six (67%) had hypokalemia, and five (56%) had mild hypochloremia. Only three dogs (33%) had lactic acidosis (Table 3).

The coagulation parameters results, of each dog, measured at the time of admission, are summarized in Table 4.

As reported in Table 4, all the dogs had at least three or more coagulation parameters out of the normal ranges. Five of the dogs (56%) had all six parameters out of the reference intervals. Activated partial thromboplastin time was increased in all the dogs; PT, D-dimer, and AT III activity were altered in eight dogs (89%); and FDPs and fibrinogen tested higher than normal in seven (78%) dogs. The FDPs, aPTT, and D-dimer were severely or very severely altered in many dogs (Table 4).

The complete blood count results showed leukopenia in four dogs (Nos. 1, 6, 7, and 9), and mild anemia in dog No. 5 (Table 5).

A correlation analysis showed a significantly high positive correlation between aPTT and clinical score (r = 0.88 and *p* = 0.001). The test did not reveal any other statistically significant correlations between the clinical score and coagulation parameters.

All the dogs survived until discharge from the VTH, except dog No. 6, suffering from SIRS and with a clinical score of 8, who died during the hospitalization.

## 4. Discussion

The results of the present study show that dogs with CPE have coagulation disorders detectable by the measurement of standard coagulation parameters. In fact, all nine dogs showed the alterations of three or more standard coagulation tests.

Blood clot disorders have been previously reported in dogs with CPE [6,7]. Otto et al. [6] described a state of hypercoagulability based on altered TEG variables, increased fibrinogen concentration, aPTT, and FDPs and a reduction in AT III activity. However, Otto’s study did not identify elevations of D-dimer in any dog, although five thrombotic events occurred. The authors concluded that D-dimer could not detect a hypercoagulable state because of the limited sensitivity of the assay that was used [6]. Whitehead et al. [7] focused primarily on TEG alterations as well as on fibrinogen and AT III activity at admission and after fluid resuscitation. They demonstrated that the hypercoagulable state was enhanced by fluid therapy. However, the measurements of additional coagulation tests such as PT, aPTT, D-dimer, and FDPs were not performed in that study [7]. In the present report, all the dogs presented a mild to very severe aPTT increase. The results evidenced a significant positive correlation between aPTT and clinical score, and this relationship could be related to the systemic inflammation. Several studies have demonstrated a close link between inflammation and coagulation [17,18,19,20,21]. This correlation is thought to intersect three different processes: (1) coagulation activation, (2) downregulation of natural anti-coagulants, and (3) inhibition of fibrinolysis. During inflammation, immune cells can activate coagulative cascades through the expression of the tissue factor (TF), resulting in activation of the extrinsic pathway, and, through the release of extracellular neutrophil traps, resulting in activation of the factor XII and the intrinsic pathway [22,23]. A study in 2009 identified a direct correlation between activated clotting time, AT, aPTT, and C-reactive protein in critical patients [24]. The aPTT is more sensitive to subtle changes in coagulation factor activity than PT. In dogs, the factor VII, which depends on PT, has a higher concentration than the other factors and requires more time before its consumption becomes evident, by a prolongation of PT [25]. Based on this consideration, we hypothesized that the low sensitivity of PT could explain why, in the present study, it was impaired in a lower number of dogs than aPTT (78% and 100%, respectively). Moreover, aPTT showed a severe or very severe alterations in five dogs (56%), while PT presented a severe or very severe alterations only in two dogs (29%). The release of inflammatory cytokines and expression of TF might lead to a reduction in endogenous anticoagulants such as AT III [20,26]. Decreased AT III activity has been associated with clinical hypercoagulability in dogs and humans and is thought to be an important contributing factor to the development of a hypercoagulable state in dogs, along with protein-losing enteropathy and nephrotic syndrome [27,28,29]. In dogs, the risk of thrombosis is thought to be moderate when AT III activity is between 50% and 75% and extreme when AT III activity falls below 50% [27]. In the present study, AT III was reduced in eight dogs (89%), with three of them (38%) having AT III below 50% and one having AT III below 75%. In addition to the effect of proinflammatory cytokines, the reduced AT III activity in dogs with CPE is likely due to other causes, including the increased loss through the damaged intestinal wall and its consumption because of endotoxin-mediated activation of coagulation, and disease progression [6,7]. Seven dogs (78%) had a moderate to very severe increases in fibrinogen concentration, which was consistent with previous studies [6,7]. In the dogs included in this study, the hyperfibrinogenemia was probably due to inflammation, as fibrinogen is an acute positive phase protein. An increase in fibrinogen is considered to be a risk factor for thrombosis because it represents the substrate necessary for the formation of a clot [6,25]. Excessive activation of the coagulation system, with subsequent fibrin clot formation and plasmin activation, results in increased production of FDPs and D-dimer [30]. In this study, D-dimer concentration was high in eight dogs (89%), and, in five of them (56%), the increase was considered to be severe and very severe. In human medicine, the D-dimer test has been used successfully in the evaluation of people suspected of having deep-vein thrombosis [31,32]. Moreover, the normal concentration of D-dimer is a valid negative predictor allowing for the exclusion of a thromboembolic disease in people [33,34]. In veterinary medicine, several reports have suggested that D-dimer might be used to detect hypercoagulability before the development of an overt thrombus. Studies reported elevated D-dimer concentration in dogs with diseases associated with a hypercoagulable state, including neoplasia, adulticide heartworm treatment, and extra-hepatic biliary obstruction [35,36,37,38]. However, it remains controversial if an increased concentration of D-dimer is directly indicative of a hypercoagulable state and thrombosis [39]. In our study, seven dogs (78%) showed high level of FDPs, and six of the dogs (86%) evidenced a concomitant increase in D-dimer. A positive FDP and D-dimer result is routinely used in conjunction with other hemostatic abnormalities to identify dogs with possible DIC [40,41,42]. Based on this assumption, if we consider the alterations of several coagulation parameters, including increased aPTT, FDPs, and D-dimer, six of the nine dogs (67%) with CPE could be considered to have hyperfibrinolysis related to DIC. In the present study, no dogs showed thrombocytopenia enhancing the early stage of DIC. Four of the nine dogs (44%) had leukopenia. Leukopenia is due to destruction of hematopoietic progenitor cells in the bone marrow, depletion of lymphoid tissues, and consumption of peripheral neutrophils due to massive demand in the inflamed gastrointestinal tract [1,2,3]. The presence of cytopenia during CPE can be useful for predicting the outcome [43]. Venous blood gas revealed metabolic acidosis in eight dogs (89%), with a decrease in electrolytes. Metabolic acidosis, hypokalemia, hyponatremia, and hypochloremia are common findings in dogs with CPE resulting from vomiting and diarrhea [44,45,46]. Three dogs presented lactic acidosis, which is consistent with the results of a recent study [47].

This study had several limitations, and the main one was the small sample size. The number of the dogs included depended exclusively on the duration of the study (3 months). During the study period, only nine dogs met the inclusion criteria. This important flaw may have affected the statistical power and limited the inferences of the results. Another limitation was the absence of a control group consisting of healthy puppies, which would have allowed for the assessment of the degree of coagulation alteration compared to age-, breed-, and body-weight-matched dogs. A further limitation was that all the possible concomitant infectious and non-infectious diseases that could have altered the hemostatic parameters were not excluded. Finally, another flaw of the study was the lack of serial coagulation tests, which would have been useful to assess the relationship between coagulation and the clinical evolution of the disease and to determine which coagulation test could be used as a prognostic indicator in dogs with CPE.

## 5. Conclusions

This study shows that dogs with CPE have coagulation disorders detectable by the measurement of standard coagulation parameters; moreover, it evidences a linear relationship between aPTT and clinical score, underlining the importance of assessing the coagulation parameters for all the inflammatory conditions. Longitudinal studies, carried out on large sample sizes, are needed to estimate the real prevalence of coagulation disorders in dogs with CPE and to understand the prognostic value of these alterations in canine parvovirus infections.

## Figures and Tables

**Table 1:** Clinical scoring system used to quantify the disease severity in dogs with parvoviral enteritis [14].

Scoring Categories	Numerical Score			
	0	1	2	3
General attitude	Normal	Mild to moderate depression	Severe depression	Collapsed or moribund
Appetite	Normal	Voluntarily eats small amounts	No interest in food	Not applicable
Vomiting	Absent	Mild, once per 12 h	Moderate, 2–5 times per 12 h	Severe, ≥6 times per 12 h
Feces	Well-formed	Soft or pasty	Watery but no bloody diarrhea	Watery, bloody diarrhea

**Table 2:** Age, sex, body weight, breed, clinical score, and presence of systemic inflammatory response syndrome in dogs with canine parvovirus enteritis.

Dog No.	Age (Months)	Sex	BW (Kg)	Breed	Clinical Score	SIRS
1	7	M	24	Crossbred	8	Yes
2	2	M	3	Crossbred	5	Yes
3	2	M	3	Crossbred	5	No
4	6	F	21	Crossbred	4	No
5	3	M	4	Pit Bull	5	No
6	3	M	6	Crossbred	8	Yes
7	6	F	8	Crossbred	7	Yes
8	8	M	21	AmStaff	6	No
9	3	F	6	Crossbred	10	Yes

M, male; F, female; BW, body weight; SIRS, systemic inflammatory response syndrome.

**Table 3:** Blood gas analyses results of 9 dogs with canine parvovirus enteritis.

Parameter	RI	Dog No.									Mean (SD)	Median (Range)
		1	2	3	4	5	6	7	8	9		
pH	7.32–7.45	7.54 *	7.39	7.38	7.41	7.45	7.40	7.36	7.43	7.36	7.41 (0.05)	7.40 (7.36–7.54)
pCO_2_ (mmHg)	33–50	18.3 *	28.7 *	38.4	26.2 *	24.6 *	40.0	19.8 *	30.6 *	43.1	30.0 (8.9)	28.7 (18.3–43.1)
HCO_3_ (mmol/L)	18–26	16.0 *	17.6 *	23.0	17.0 *	17.6 *	25.5	11.4 *	20.8	24.6	19.3 (4.6)	17.6 (11.4–25.5)
Lac (mmol/L)	0.5–2.0	4.0 *	0.6	0.6	0.9	0.6	2.0	4.4 *	0.7	3.6 *	1.9 (1.6)	0.9 (0.6–4.4)
Na (mmol/L)	140–150	137 *	133 *	132 *	137 *	126 *	138 *	144	139 *	144	137 (6)	137 (126–144)
K (mmol/L)	3.9–4.9	3.3 *	3.8 *	3.7 *	4.0	4.6	3.0 *	4.1	3.4 *	3.0 *	3.6 (0.5)	3.7 (3.0–4.6)
Cl (mmol/L)	109–120	105 *	111	106 *	110	109	107 *	114	108 *	107 *	108 (3)	108 (105–114)
Ca (mmol/L)	1.25–1.50	1.06 *	1.26	1.27	1.26	1.13 *	1.27	1.09 *	1.19 *	1.33	1.20 (0.09)	1.26 (1.06–1.33)
AG (mmol/L)	12–20	19.2	7.9 *	7.4 *	13.2	3.8 *	8.9 *	23.1 *	13.2	16.0	12.5 (6.2)	13.2 (3.8–23.1)
BE (mmol/L)	−2/+2	−6.6 *	−7.5 *	−2.3 *	−7.7 *	−6.5 *	−0.6	−14.1 *	−3.5 *	−1.0	−5.5 (−4.2)	−6.5 (−14.1–0.6)

pCO_2_, partial pressure of carbon dioxide; HCO_3_, bicarbonate; Lac, lactate; Na, sodium; K, potassium; Cl, chloride; Ca, calcium; AG, anion gap; BE, base excess; RI, reference intyervals; SD, standard deviation. * Values out of reference intervals.

**Table 4:** The coagulation parameter results and the degree of alteration for nine puppies affected by canine parvovirus enteritis.

Parameter	RI	Dog No.									Mean (SD)	Median (Range)
		1	2	3	4	5	6	7	8	9		
PT (s)	6.7–8.2	9.1 *	9.1 *	8.7 *	8.1	8.8 *	17.9 ****	8.5 *	7.6	16.0 ***	10.4 (3.8)	8.8 (7.6–17.9)
aPTT (s)	10.6–12.8	21.9 ****	19.9 ***	17.1 **	15.7 *	15.4 *	20.6 ***	21.7 ***	18.5 **	37.4 ****	20.9 (6.6)	20 (15.4–37.4)
Fib (mg/dL)	150–400	901 ****	318	968 ****	559 **	588 **	120	576 **	549 **	588 **	574 (259)	576 (120–968)
AT III (%)	100–148	94 *	41 ***	90 *	125	90 *	18 ***	36 ***	97 *	71 **	73 (35)	90 (18–125)
D-dimer (µg/mL)	0.01–0.33	0.40 *	1.32 ****	0.38 *	0.33	1.19 ****	0.84 ****	0.40 *	0.50 ***	2.82 ****	0.90 (0.80)	0.50 (0.33–2.82)
FDPs (µg/mL)	0–2.5	4.1 ***	4.0 ***	5.6 ****	2.7 *	5.3 ****	1.3	4.0 ***	2.3	6.0 ****	3.9 (1.6)	4.0 (1.3–6.0)
Total altered parameters		6	5	6	3	6	4	6	4	6		

PT, prothrombin time; aPTT, activated partial thromboplastin time; Fib, fibrinogen concentration; AT III, antithrombin activity; FDPs, fibrinogen degradation products concentration; RI, reference intervals; SD, standard deviation. * Mild alteration (<25%); ** moderate alteration (≥25 and <50%); *** severe alteration (≥50% and <100%); **** very severe alteration (≥100%).

**Table 5:** The complete blood count results of 9 puppies affected by canine parvovirus enteritis.

Parameter	RI	Dog No.									Mean (SD)	Median (Range)
		1	2	3	4	5	6	7	8	9		
RBC (10^12^/L)	5.5–8.5	7.4	6.5	6.8	6.5	4.5 *	6.7	7.0	6.9	6.9	6.6 (0.8)	6.8 (4.5–7.4)
Htc (%)	39–56	53	46	44	46	27 *	43	48	49	46	45 (7)	46 (27–53)
Hb (g/L)	110–190	150	126	118	126	74 *	129	127	141	123	124 (21)	126 (74–150)
MCV (fL)	62–72	72	70	65	70	61 *	64	68	72	66	68 (4)	68 (61–72)
MCH (pg)	20–25	20.3	19.4 *	17.4 *	19.4 *	16.6 *	19.3 *	18.0 *	20.5	17.8 *	18.7 * (1.3)	19.2 (16.6–20.5)
MCHC (g/L)	300–380	282 *	276 *	267 *	276 *	272 *	301	265 *	285 *	270 *	277 * (11)	276 (265–301)
RDW (%)	11–15.5	14.1	14.3	15.5	14.3	16.4 *	15.1	12.9	15.3	14.4	14.7 (1)	14.4 (12.9–16.4)
PLT (×10⁹/L)	117–460	357	159	424	159	153	439	214	171	394	274 (126)	214 (153–694)
WBC (×10⁹/L)	6.1–17	0.5 *	6.2	8.3	6.2	10.6	0.4 *	2.3 *	14.8	0.5 *	5.5 (5.0)	6.2 (0.4–14.8)

RBC, red blood cells; Htc, hematocrit; Hb, hemoglobin; MCV, mean corpuscolar volume; MCH, mean corpuscolar hemoglobin; MCHC, mean corpuscolar hemoglobin concentration; RDW, red cells ditribution width; PLT, platelets; WBC, white blood cells; RI, reference intervals; SD, standard deviation. * Values out of reference intervals.

## Data Availability

Not applicable.
